# Modelling the Effect of a Functional Endothelium on the Development of In-Stent Restenosis

**DOI:** 10.1371/journal.pone.0066138

**Published:** 2013-06-13

**Authors:** Hannan Tahir, Carles Bona-Casas, Alfons G. Hoekstra

**Affiliations:** Computational Science, Informatics Institute, Faculty of Science, University of Amsterdam, Amsterdam, The Netherlands; University of Adelaide, Australia

## Abstract

Treatment of stenosed coronary arteries by balloon angioplasty and stenting results in arterial injury including severe damage to the endothelium at the site of treatment and initiates a complex cascade of inflammatory processes that may lead to the development of in-stent restenosis (ISR). Many clinical and biological factors involved in the progression of restenotic lesions have been studied in detail over the past few years but the mystery behind the pathophysiological mechanisms of this disease is still unresolved. In the present work, the effects of re-endothelialization and nitric oxide release on neointimal growth are investigated *in-silico* using a two dimensional multi-scale model of ISR. The effect of stent deployment depths on the development of ISR is studied as a function of time after stenting. Two dimensional domains were prepared by deploying bare metal stent struts at three different deployment depths into the tissue. Shear stress distribution on endothelial cells, obtained by blood flow simulations, was translated into nitric oxide production that keeps the smooth muscle cells in quiescent state. The cellular growth trends were plotted as a function of time and the data indicate a positive correlation between the neointimal growths and strut deployment depths in the presence of a functional endothelium, in qualitative agreement with in-vivo data. Additionally, no ISR is observed if a functional endothelium appears much earlier.

## Introduction

Coronary heart disease (CHD) remains a life-threatening complication with a high mortality and morbidity rate [1]. The main cause of CHD is the development of atherosclerotic plaque that causes an occlusion or stenosis inside the arteries and leads to a decrease in the blood flow. Patients suffering from CHD are usually treated by balloon angioplasty and stent placement. Endovascular stents are placed during the angioplasty procedure to prevent the vessel from collapsing or closing up again. A re-growth of the tissue within the stented part of the artery is known as in-stent restenosis (ISR). Developments in the field of endovascular stents have substantially reduced the rates of ISR from 30% (with the use of bare metal stents) to 10% (with drug eluting stents) [2]. But the chance of developing a restenosis varies between patients, depending on age and health condition, vessel size, and the complexity of the developed lesion. There seems to be a correlation between the arterial injury due to stent deployment and the degree of restenosis [3], [4], [5] but there is no significant injury score information available for cases where restenosis did not develop. Such experiments are required to analyze whether the injury is the only key factor initiating this response or if there may be some other unknown factors. In this study, we aim to use the results obtained from an *in-silico* multi-scale model of ISR to identify processes that inhibit the development of restenosis, specifically focusing on the role of re-endothelialization. The innermost layer of a vessel, the endothelium, consists of a mono-layer of endothelial cells (ECs). ECs play an important role in regulating the vascular tone and permeability by managing the exchange of molecules in response to physical and chemical signals [6], [7]. ECs sense fluid stresses and regulate their effects by releasing vasodilators and vasoconstrictors to the underlying SMCs. In the absence of a healthy and intact endothelium, the balance between the vasodilators and vasoconstrictors is disturbed and a mismanagement of the vascular tone occurs that can lead to the development of plaque [8], [9]. After injury caused by balloon angioplasty or stent deployment in percutaneous coronary intervention, the endothelium is partially or completely denuded [6], [10], triggering inflammatory mechanisms like platelets aggregation and formation, smooth muscle cells (SMCs) migration and proliferation, extra cellular matrix (ECM) formation and, finally, ISR [2]. The presence of stent inside the artery influences the flow dynamics and induces flow re-circulation and stagnation zones around the stent struts [11], [12], [13].

In physiological conditions, endothelial cells are exposed to flow shear stress whereas SMCs in the medial layer are usually subjected to the cyclic strain caused by the pulsatile nature of blood flow [14]. Medial SMCs are also exposed to very low levels of interstitial flow driven by the transmural pressure [15]. Right after the endothelium damage following stent deployment, the superficial layer of SMCs is exposed directly to the flow shear stress. However, the direct link of the flow shear stress and elevated levels of interstitial flow on the phenotypic changes in the SMC still remains controversial [16], [17]. In normal conditions, SMCs express contractile phenotype and remain quiescent. The natural wound healing process in response to injury involves the production of several growth factors, e.g. platelet-derived growth factor, vascular endothelial growth factor, insulin like growth factor, fibroblast growth factor etc. [18]. Medial SMCs de-differentiate into proliferative synthetic phenotype after getting exposed to these growth factors and inflammatory mediators [19]. The phenotypic changes in SMC in response to cyclic strain have also been extensively studied over the past few years, but its effect on the SMC growth varies between the species, their location in the vascular bed and the presence of ECM [20]. In the present study, we do not take into account the effects of cyclic strain and direct exposure of shear stress on SMCs.

It has previously been reported that the presence of an intact endothelium is sufficient to promote the inhibition of the underlying medial SMCs proliferation [21]. However, it has also been observed that re-endothelialization appears rapidly after stent deployment [22] but most of the time, the newly regenerated endothelial layer is dysfunctional [21]. The phase of an abnormal regulation of these mechanisms due to the failure of ECs to perform their typical functions is known as endothelial dysfunction [23].

A functional endothelium senses shear stress and translates that as a stimulus to produce nitric oxide (NO). NO, being a highly diffusible molecule and a predominant mediator to control the vascular function, is generated by one of the isoforms of the nitric oxide synthase enzyme (NOS) [24]. Endothelial NOS (eNOS) is produced by the endothelial cells. The amount of eNOS released by ECs depends on the availability of intracellular calcium (Ca^2+^) that is regulated in response to shear stresses and blood borne agonists activated pathways, such as thrombin, adenosine nucleotides, acetylcholine, bradykinin etc. [7], [25], [26]. Recent evidence [27] also suggests that fluid shear stress modulates the NO production through platelet endothelial cell adhesion molecule (PECAM-1) which directly regulates the basal eNOS activity. And, in agreement with [28], recent experiments have measured PECAM-1 expression and have shown that a regenerated endothelium does not necessarily translate into a functional endothelium [29].

In the current paper, we report the importance of re-endothelialization and its possible effects on the development of neointimal lesion. The outcome of stent deployment, using a bare metal stent, by varying the deployment depth is studied. Moreover, the importance of the presence of a functional endothelium is highlighted by showing results produced by our two-dimensional multi-scale model of ISR [30], [31]. Results are also compared with in-vivo data reported in a previous paper [32]. The study finally discusses the importance of promoting early appearance of functional endothelium by showing that in that case the model results in no restenosis at all regardless of the degree of deployment depth. The paper also discusses limitations involved in the present work and highlights possible future improvements in the tissue model by adding more biological processes.

## Materials and Methods

An *in-silico* two dimensional multi-scale model of in-stent restenosis based on porcine coronary artery data has been developed, involving blood flow and SMCs proliferation [30], [31]. The blood flow and SMC models are coupled together using the MUltiScale Coupling Library and Environment (MUSCLE) [33], [34].

### Artery Generation and Stent Deployment

A small two-dimensional longitudinal section of a healthy porcine coronary artery, considering a segment length of 1.5 mm and width of 1.24 mm, is used as benchmark geometry. Five layers of densely packed SMCs are generated, with an average radius of 15 µm, to achieve a vessel thickness (the medial layer) of 120 µm. The lumen width is set to 1 mm. A thin layer of internal elastic lamina (IEL) agents is created inside the lumen on top of the SMCs in order to maintain a barrier between the blood flow and the SMCs. During balloon angioplasty, the pressure exerted by the balloon on the arterial wall damages the endothelial layer and subsequent stenting results in further damage to the wall, e.g. breaking of the IEL. We assume that due to the balloon angioplasty, the endothelium is completely denuded. Moreover, stent deployment ruptures the IEL layer from the sites where struts are deployed. This rupture of IEL is used as a surrogate of injury caused by the strut to the vessel wall. The deeper a strut penetrates into the tissue; more the IEL is ruptured. The ruptures in the IEL allow the SMCs from the medial superficial layer to come into contact with the flow shear stresses. The SMCs that are directly exposed to flow have the tendency to change their phenotype from a quiescent contractile state to a proliferative synthetic state. Stent deployment is simulated by pushing two square bare metal stent (BMS) struts into the vessel wall. The deployment process is modelled by computing forces on each cell, including a hoop stiffness calculated as a function of radial displacement from the initial cell position. The removal of IEL elements during the deployment is modelled based on the longitudinal and hoop stresses [31].

### Blood Flow Model

After stent deployment, the model proceeds by repeatedly computing blood flow in the lumen, obtaining wall shear stresses (WSS) that are used in the model for SMC proliferation. Migration and proliferation of SMC changes the lumen geometry, which is then sent to the blood flow model that uses a standard BGK Lattice Boltzmann method (LBM) to simulate steady state flow within the domain, assuming blood as a Newtonian and incompressible fluid with a constant viscosity, *µ* = 4 mPa.s, density *ρ* = 1000 Kg/m^3^ and *Re* = 120 [35], [36], [37]. In order to run the LBM flow solver, the initial domain after stent deployment that contains information about cell center positions and their radii, is converted into a computational mesh. Periodic boundary conditions are imposed at the inlet and outlet whereas a Dirichlet boundary condition for the velocity in combination with a bounce-back rule is enforced on the walls. The flow simulation calculates shear stress values on the vessel boundaries and then this parameter is mapped back from a lattice grid onto the individual cells in the SMC model. As the SMCs proliferate and the vessel wall is occluded, resistance in the vessel will increase mostly due to the decrease of the lumen cross sectional area (for a more detailed study on vessel resistance due to stenosis the reader is referred to [38], [39]). However, despite mild or moderate stenosis, coronary blood flow is maintained by compensatory vasodilatory regulation of the microcirculation (autoregulation). In fact, resting coronary blood flow remains constant until epicardial luminal narrowing exceeds 85 to 90% diameter. Unlike resting flow, maximal hyperemic coronary blood flow is attenuated when diameter stenosis approaches 45 to 60% [40]. We will assume that blood flow remains constant for the whole restenotic process. This will imply that a decrease in the vessel diameter will translate into a quadratic increase in the blood velocity and will therefore increase the WSS values.

### SMC Model

The shear stress values on each cell are required as an input for the SMC model [31]. The SMC model is an agent-based model (ABM) where the vascular tissue is modelled as a collection of SMCs. Each SMC is individually modelled as an agent. The model contains two solvers: (i) a physical solver that computes new equilibrium positions of cells based on mechanical cell-cell attraction, cell-cell repulsion and frictional forces. (ii) a biological solver that controls the proliferation of SMC in response to a set of biological rules. The biological state of each SMC is assessed at every iteration. The progression of each agent is regulated by a cell cycle that has three stages. G0 a quiescent state where SMCs do not proliferate and show a contractile phenotype; G1 a growth phase where a cell gradually increases its size and S/G2/M is the final stage where a cell further divides into two daughter cells. During the execution of the biological solver, SMCs may remain in the quiescence state or may change into a proliferative stage based on the rules defined within ABM. And vice-versa, proliferative SMCs may change their phenotype and return to the quiescent contractile stage. Further details about the model itself are presented elsewhere [30], [31].

As an extension to the above described ISR model, we added the process of re-endothelialization to investigate its possible role in inhibiting the development of neointimal hyperplasia.

**Figure 1:**
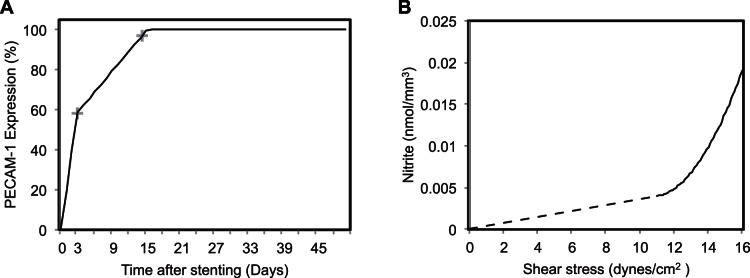
Presence of PECAM-1 expression and relationship between shear stress and Nitrite concentration. (A) Presence of PECAM-1 expression as a function of time after stenting. Black solid line is interpolated from the two data points (marked with**+**symbol) by Nakazawa et al. 2010. [29]. (B) Relationship between wall shear stress and Nitrite concentration. Black dashed line is interpolated from Guo et al. 2009 whereas the black solid line represents the actual data shown by Guo et al. 2009 [42].

**Figure 2:**
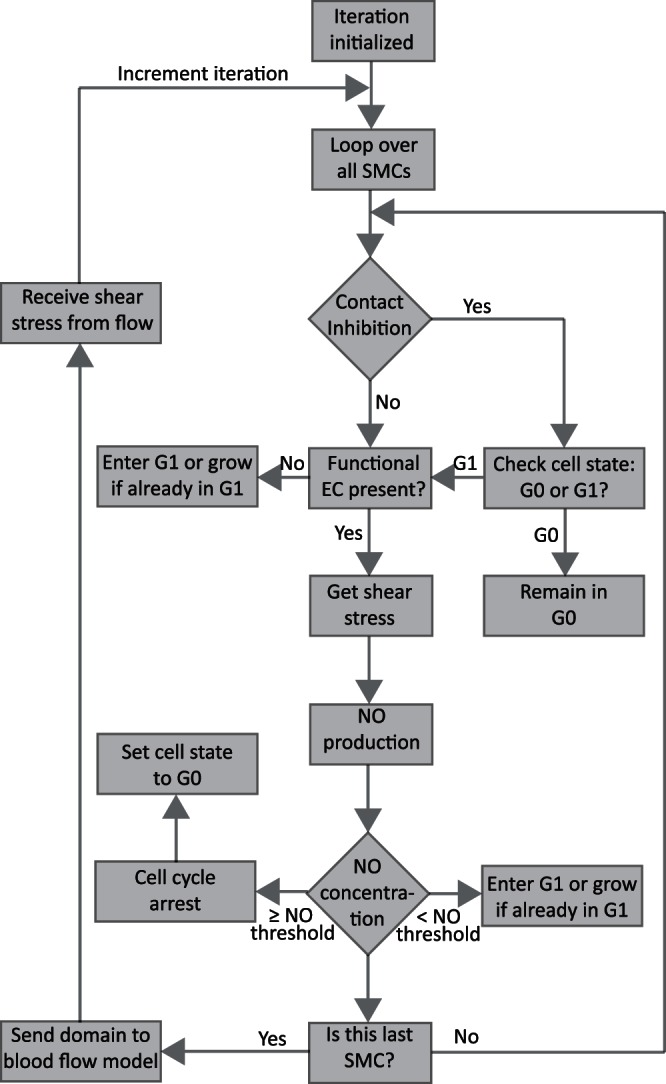
SMC regulation flow chart.

In the current model, ECs are not explicitly modelled. Since endothelium is made up of a thin monolayer of endothelial cells and the height of ECs is small in comparison to SMCs, we assume that blood flow is not affected by this thin cell layer. Therefore, shear stress acting on the inner most layers of SMCs is considered to be the same as for the endothelial cells layer. Nakazawa et al. [29] measured PECAM-1 expression released by mature (functional) endothelial cells after stenting. Their data only consists of two data points at 3 days (59% PECAM-1) and 14 days (94% PECAM-1) post stenting. We have made a rough extrapolation to be able to use that data at each time step, which in our case is one hour for SMC model. We used the percentage of PECAM-1 expression shown in Figure 1A. as an estimation of the presence of a functional endothelium. It is clear from Figure 1A. that approximately 59% of functional endothelium was present on the 3^rd^ day after stenting, furthermore this expression turned into 100% after 15 days. So a probability function based on PECAM-1 expression (Figure 1A), representing the existence of a healthy endothelium, is used. We cover the domain with the appropriate percentage of functional endothelium and we incrementally add more ECs at each time step according to probabilities derived from Figure 1A. Next the shear stress sensed by those endothelial cells regulates the production of Nitrite, a stable metabolite of NO and a marker of local NO production [41], using experimental results shown by Guo et al. [42] in Figure 1B. Guo et al. [42] only presented the shear stress to nitrite conversion data between shear stress values ranging from 9 dyne/cm^2^ to 16 dyne/cm^2^. So, we extrapolated it to 0 dyne/cm^2^, assuming that no shear stress produces no nitrite. WSS was directly applied to SMCs in our previous model [32] because there was no endothelium present at that time, whereas in the current model, SMCs are only allowed to respond to NO concentration that is being produced by the functional ECs in response to WSS. The NO produced then serves to inhibit the growth and proliferation of the underlying SMCs by causing a cell cycle arrest in the G1 stage and prevent cells to enter into S-phase [21], [43], [44]. Our model of regulation of SMCs in response to shear induced NO production is shown in Figure 2, where the progression of every SMC within the cell cycle is controlled by contact inhibition and an NO concentration rule. NO concentration rule is a threshold-based rule applied to each SMC, where, if the NO sensed by that specific SMC (obtained from the functional endothelium) is higher than a predefined threshold, then it causes a full cell cycle arrest in the SMC. We don’t consider a baseline nitrite or NO concentration in the current model because it will not have an effect on the SMC growth inhibition. In the current model, SMCs can only remain in a quiescent state if the NO concentration on a cell exceeds a predefined threshold. The NO threshold of 1 µM (0.001 nmol/mm^3^), taken from Coneski et al. 2012 [45], is used.

A typical cell cycle length of aortic porcine SMCs is 32 hours [46]. In our model, once a cell enters into G1 stage, it can only grow and remain in G1 if the NO concentration is lower than the predefined threshold. The cell can only enter into S/G2/M phase if it was allowed to grow in the G1 phase for 16 hours. Once entered into the S/G2/M phase, no more rules are applied on the cell, which proceeds further to divide into two daughter cells after staying for another 16 hours in the S/G2/M phase.

In order to evaluate the dynamics of ISR in the presence of a functional endothelium, taking into account the injury scores caused by different penetrations of struts into the tissue, we must know when the endothelium recovers from the arterial injury caused by the stent and when does it become functional. The data interpolated from Nakazawa et al. [29] provide us a good approximation of the time course of a functional endothelium regeneration or recovery (figure 1A). The endothelium recovery time course shown by Nakazawa et al. [29] is an important finding, but on the other hand, this exact recovery rate or time frame might only be true for a certain number of animals and cannot be extended to the whole population belonging to that group. Therefore different scenarios of endothelium recovery rates are investigated in this study. Three cases are modelled in order to investigate the effect of functional endothelium recovery rate on the restenotic lesion development. These cases are explained in Table 1.

**Table 1:** Three cases, based on different endothelium recovery rates, are used to investigate the presence of functional endothelium towards the development of ISR.

Cases	Description
Case-1	59% endothelium present after day 3^rd^ post stenting and endothelium recovery is 100% after 15 days post stenting. Data is interpolated from Nakzawa et al. [29]. The endothelium recovery rate is 3.42% per day after day 3^rd^.
Case-2	The initial endothelium recovery is the same as case-1 (59% endothelium present after day 3^rd^ post stenting) but the complete recovery of the functional endothelium is delayed by one week (23^rd^ day post stenting), resulting in a slower recovery rate after day 3^rd^. The endothelium recovery rate in this case is 2.05% a day after day 3^rd^.
Case-3	0% endothelium present immediately after the stent deployment and a linear endothelium recovery is assumed, turning into 100% after 23 days post stenting. In this case, we assume a severe injury of the endothelium compared to case-1 (no fast recovery of endothelium present before day 3^rd^), but a rather high overall recovery rate, 4.35% a day.

The effect of strut deployment depths on the neointimal growth is also evaluated for each case by deploying struts at three different depths (90 µm, 110 µm and 130 µm). Each simulation was run 10 times to obtain an average neointimal area. Neointimal area is calculated as a sum of all the surface areas occupied by the neointimal cells. We calculate the area occupied by the initial cells at the start of the simulation (after stent deployment) and then we subtract this initial area from the total cell area measured at a certain time point to obtain neointimal cell area. It is also important to note that a complete removal of endothelium and a partial rupture of IEL (based on the deployment depth) are assumed immediately after the stent deployment. The percentage of endothelium used in each case represents the presence of functional endothelial cells only. We do not take into account the presence of dysfunctional endothelial cells in the current model.

In all cases, NO is not modelled explicitly and once generated in response to shear stress is assumed to quickly diffuse into SMCs. However, in reality, NO has a very short life time (seconds) and once generated by endothelial cells, it produces cyclic guanosine monophosphate (cGMP), by diffusing into the vascular SMCs [47]. In our model, we take an assumption that the overall NO produced by ECs is directly diffused into the underlying SMCs. This might be an over simplification of this complex chain of processes but the main idea of the current study is to explore the effects of re-endothelialization and regulation of SMCs by means of NO to produce similar neointimal growth trends as observed in the in-vivo data.

## Results

**Figure 3:**
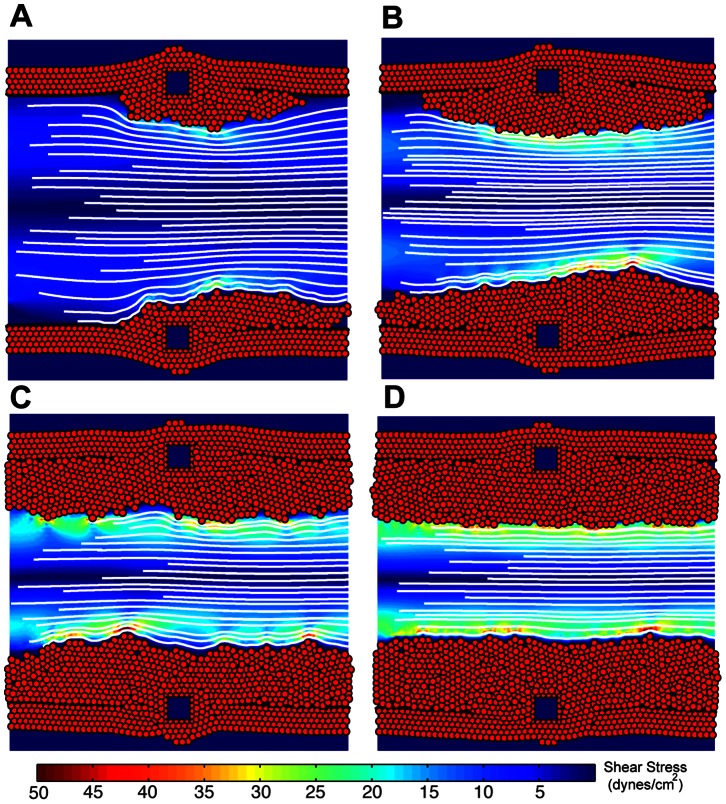
Neointimal growth after 50 days post stenting, showing SMC growth and flow streamlines. Struts were deployed at a depth of 110 µm. (A) Case-1 where NO along with EC probability effective from day 3rd after stenting and 100% after 15 days, (B) case-2 where NO effective from day 3rd and 100% after 23 days, (C) case-3 where 0% endothelium was present at day 0 and 100% after 23 days and (D) results from our previous model in order to compare the effect of re-endothelialization on SMC growth.

A qualitative comparison of the simulated ISR between three cases is shown in Figure 3 where neointimal cell growth is visualized 50 days post stenting. Figure 3A represents the case-1 where ECs with a certain probability (approximately 59%) were present from the 3rd day after stenting and this probability was 100% after 15 days, showing very little neointimal cell growth (almost no restenosis). Asymmetry between the left half and the right half seems to be present in figure 3A and that is mainly due to WSS. Low WSS is usually observed distal to the stent struts as compared to the proximal side of the struts. Since endothelium appears much faster in case-1, the asymmetry is more prominent. A slightly higher amount of neointimal growth, shown in Figure 3B, is observed assuming a marginally delayed healing of the endothelium (case-2). Figure 3C illustrates a dramatic increase in the neointimal cell growth with the assumption of a very slow and delayed endothelium recovery where 0% endothelium at day 0 and 100% mature endothelial cells present within the domain after 23 days (case-3). Asymmetrical proliferation proximal and distal to stent struts is not prominent in case-2 and case-3. Figure 3D shows results from our previous model without considering the effect of re-endothelialization. There is a remarkable qualitative difference in terms of cell growth in all cases (figure 3).

**Figure 4:**
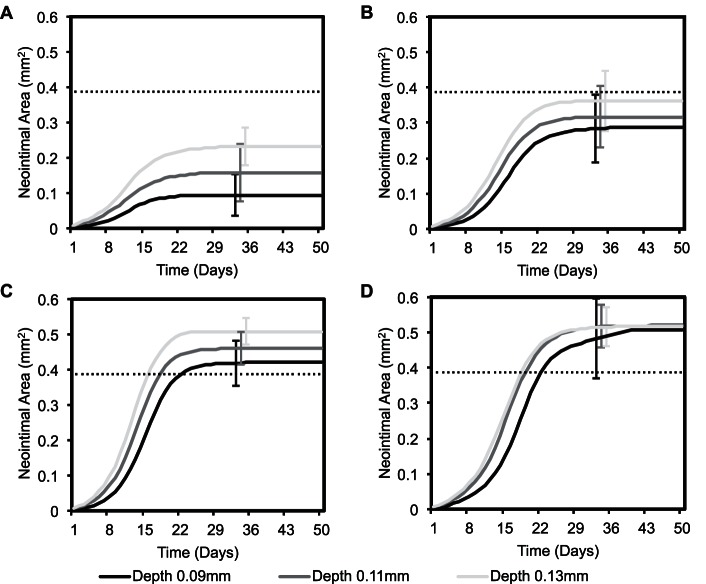
Comparison of results showing neointimal area as a function of time in all cases. (A) case-1, (B) case-2, (C) case-3 and (D) previous model outcome. An average result over 10 simulations is plotted for every deployment depth in each case while error bars represent the standard deviation. Black dotted line in all cases represents the 50% lumen occlusion point according to our domain dimensions.

In order to understand the effect of deployment depth in all three cases, we plotted neointimal area as a function of time for all deployment depths (Figure 4). Some studies define ISR as a percentage of lumen occlusion from its initial diameter after stent deployment, where if the lumen is occluded more than 50%, it is referred as ISR [48], [49]. So in Figure 4, we used this definition as a reference point to characterize all three cases that either they produce ISR or not. It is quite clear from figure 4D that our previous model had a limitation of reaching to more or less same level of neointimal growth regardless of the degree of deployment depth. This limitation was highlighted in our previous paper where the maximum number of neointimal cell number was almost constant at all deployment depths.

Figure 4A shows the results obtained with the assumption of case-1 where 100% ECs were present after 15 days. The growth trends from case-1 clearly indicate different endpoints and results in different neointimal area growths. Moreover, it is also evident from figure 4A that case-1 does not produce ISR as the area covered by the neointimal growth is far below the 50% lumen occlusion reference line. Figure 4C illustrates the results produced with the assumption of case-3 and shows a severe regrowth of the neointimal tissue at all deployment depths. A slightly moderate lesion development is observed in case-2 (figure 4B) where the 100% recovery was slightly delayed as compared to case-1. It is also apparent from all the three cases shown in figure 4 (A, B and C) that deeper penetration of struts result in a higher neointimal growth and vice versa.

**Figure 5:**
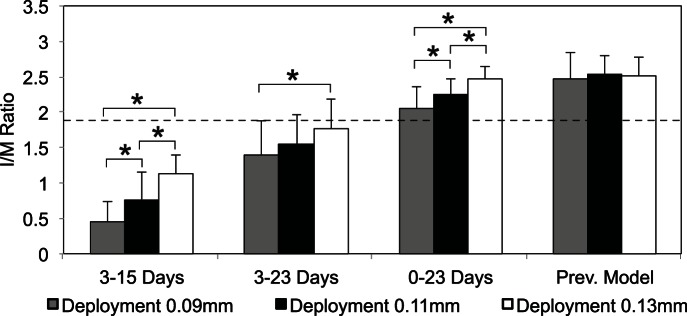
Intima/Media (I/M) ratio for all three cases and compared with previous model results. The black dashed line represents the reference line of 50% lumen occlusion. All the data are presented as mean +SD. * represents p<0.05 between the deployment depths for each case.

To further evaluate the effect of strut deployment depth on the restenotic tissue growth, the intima/media (I/M) ratio is calculated on the basis of area occupied by both layers of tissue. Figure 5 presents the mean I/M ratio calculated 50 days post stenting and it is clear that I/M ratio is directly proportional to strut deployment depth for all three cases. However, our previous model does not support this notion. It is evident from figure 5 that increase in the I/M ratio strongly depends on the injury caused by the stent struts and difference in the I/M ratio between three deployment depths is significant (p<0.05) for case-1 and case-3. However, this significant difference in case-2 is only noticeable between the growths produced with the deployment depths of 0.09 mm and 0.13 mm (figure 5).

## Discussion

**Figure 6:**
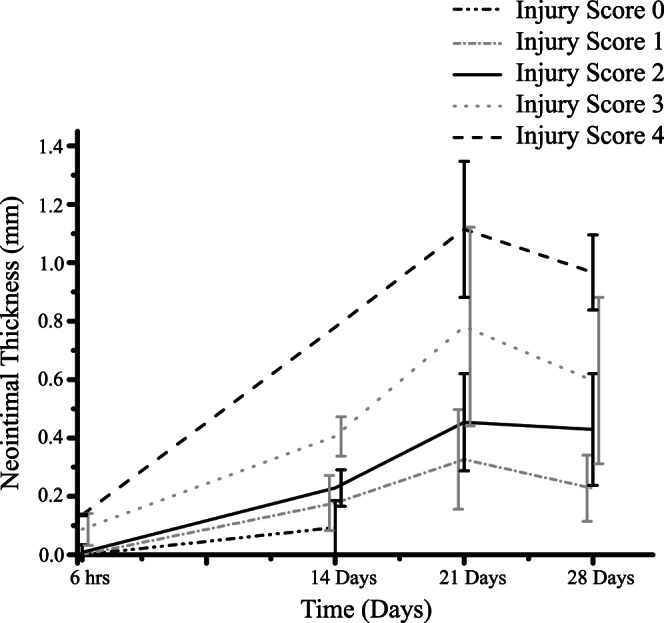
In-vivo data of stented porcine arteries, showing neointimal thickness as a function of injury score. The data from transversal sections show that neointimal thickness is directly proportional to the arterial injury at all time points after stenting. Data is measured at 6 hours, 14 days, 21 days and 28 days post stenting. Error bars are slightly shifted for the sake of visualization. This graph is reused from our previous publication (figure 6 on page 371 from Tahir et al, 2011 [32]) to allow the reader to compare our computational results with the trends seen in the in-vivo data sets.

Our results suggest an inhibition of SMC proliferation as soon as a functional endothelium is present (case-1, 2 & 3), as compared to our previous results where SMC growth was inhibited only by a high wall shear stress as a result of the narrowing of the vessel (Figure 3, Figure 4, Figure 5). Figure 4 and 5 also demonstrate a significant difference in the neointimal area and I/M ratio between all the cases, showing very little neointimal growth (almost no restenosis) with the assumption of having a 100% probability of a functional endothelium after day 15 post stenting (case-1), whereas the other two cases (case-2 & case-3) dictate the presence of a healthy endothelium after 23 days and produce restenosis based on the presence of an early functional endothelium and the endothelium recovery rate. These results hint on the importance of a fully functional endothelium by showing different neointimal cell growths. In all three cases, the lesion growth rate, dictated by the steepness of the curve (figure 4) is directly proportional to the injury caused by the stent. Moreover, the deeper a strut penetrates into the tissue, the higher the neointimal area after 50 days and this is true for all three cases shown in figure 4. The growth trends and restenotic lesion development, produced by the 2D ISR model, agree qualitatively with the in-vivo data shown in figure 6, where an increase in the deployment depth results in a faster neointimal growth, which has already been shown in our previous findings [32].

The actual model provides significant improvements compared to our previous results [32]. The previous model reproduced in a qualitative way the initial dynamics of the response, a positive correlation between the speed of the early time ISR response with deployment depth of the stent. However, the final neointimal thickness or neointimal cell number was almost constant for all the deployment depths, in clear disagreement with the in-vivo data (Figure 6). The reason why we could not capture it was because of the use of an over-simplified WSS rule. Once the shear stresses on cells exceeded a predefined threshold, the process of neointimal growth stopped. WSS is highly determined by the lumen size, as a decrease in the lumen size results in an increase in the flow velocity (in order to keep the flux constant), which results in increased friction of the peripheral blood layers on the arterial wall and therefore a higher WSS. Then, by using that rule, SMCs would proliferate until the lumen would be narrowed enough, regardless of the initial deployment depth of the stent and in contradiction with observations. The thickness of the strut also influences the damage caused by the stent to the vessel wall, resulting in a higher injury with the use of a thicker strut and vice versa. This effect has already been captured in our previous publication [32] and resembles well with other human clinical studies [50], [51]. In the current study, we captured the influence of a functional endothelium that results in different end points (Figure 3 and 4), therefore results completely agree with the in-vivo data in a qualitative manner (Figure 6).

We have hypothesized that a healthy and functional endothelial layer is necessary for the control of the neointimal formation. Once endothelium is functionally present and regulates vasodilators, it can aid to stop the SMC hyperplasia by keeping them in a quiescence state via NO release. Our results do not try to claim that endothelium presence is the only factor required to inhibit restenosis, there is a need to promote a fully functional endothelium that is considered to inhibit the growth of the underlying cells. So despite of the urgent need to recover the endothelial layer, it is much more important to identify the mechanisms that make this monolayer of EC fully functional in order to promote proper cellular signaling generated in response to physical and chemical stimuli.

Endothelial denudation after balloon angioplasty and stent deployment triggers inflammatory processes within the vessel. The natural healing response of the artery, leading to the development of ISR, continues until the re-endothelialization occurs [52]. Endothelial cells regenerate within a couple of days after the stent deployment. De Prado et al. [22] reported that complete re-endothelialization was complete within 7 days after the deployment of bare metal stents in the healthy swine coronary arteries. On the other hand, Finn et al. [53] observed similar endothelial cell re-growth after 14 days in the swine arteries using cobalt-chromium stents. Some other studies report that endothelial progenitor cells capturing stents showed better results by promoting faster endothelium recovery but this accelerated endothelialization did not seem to reduce the amount of neointimal thickness at 28 and 90 days as compared to the group of control stents deployed in porcine coronary arteries [28]. These studies were of great importance in order to estimate the time course of re-endothelialization in the swine models but none of those evaluated the functionality of newly regenerated endothelium. Some recent studies suggest that the regenerated endothelial cells after the vascular injury exhibit a selective loss of functionality [21], [54]. The dysfunctional endothelial layer results in a reduced production of NO that is not sufficient to stop the development of ISR. Given the need to analyze the functionality of endothelium in the coronary arteries, it is rather difficult to assess its function due to the invasive nature of the coronary vasculature. The measurement of its function is usually done with an infusion of Acetycholine in the intracoronary circulation to determine the endothelial dependent functionality [55]. Coronary arteries respond to the infused intra-coronary Acetycholine by causing microvascular dilation and an increase in the flow in the presence of a functional endothelium whereas a vasoconstriction occurs in the absence of a functional endothelium, resulting in a decrease in the coronary flow [25], [56]. This protocol gives a valuable estimate of endothelium functionality but it does not provide enough quantitative information. Another useful predictor to assess the presence of an intact endothelium is the PECAM-1 expression and a decrease in this expression is associated with an impaired endothelial recovery and functionality [29]. In-vitro and in-vivo experiments have shown that activation of eNOS is regulated by PECAM-1 expression [27]. Nakazawa et al. [29] measured the PECAM-1 expression as a function of time after stenting in the normal pig coronary arteries using confocal microscopy. Interpolation of the data measured by Nakazawa et al. gave us an estimation of the presence of a functional endothelium present within the vessel after stenting.

The main objective of this study is to obtain different amounts of neointima based on the different injury scores, which was the main flaw of our previous model, but it also points towards an answer to the basic question, which is why 70% of patients do not develop restenosis and why the remaining 30% tend to suffer from ISR [2]. This model allows us to estimate the overall growth of SMC by tuning just one parameter, which is availability of a fully functional endothelium. In our previous paper, we modelled the effect of a drug release from a DES using finite difference scheme and results showed a delay in the neointimal growth response by inhibiting the SMC proliferation [32]. However, current study is only limited to the use of BMS in-order to isolate the effect of endothelium restoration and its possible role in controlling the ISR development. Moreover, antiproliferative drugs used in the DES have been shown to delay the re-endothelialization by inhibiting the ECs proliferation [57], [58]. Modelling the growth of neointimal lesion along with a delayed re-endothelialization in the presence of DES will be our future area of research. We also aim to include ECM in the current model to represent a more realistic arterial tissue and to simulate the effect of ECM degradation on the neointimal development.

One of the limitations of the present model is the pathway through which NO is produced. Currently, NO is only produced in response to the WSS. However, NO in the arterial wall can also be formed under the presence of other isoforms of the enzyme NOS such as neuronal NOS (nNOS) or inducible NOS (iNOS) [9]. Future work should involve the presence of these other sources of NO and their contribution towards the inhibition of neointimal tissue growth. Another limitation of the study is that the model is currently restricted to porcine data. Regardless of the fact that porcine arteries response to injury shows similarities with the human arteries [59], we should not ignore the translational aspects from a healthy porcine vessel to what is happening in the diseased human arteries. The growth response might be different in the presence of an atherosclerotic plaque [60]. This future work will involve coupling of an atherosclerosis model with the current flow and growth models.

### Practical Applications

The development of the ISR model was motivated by the desire to develop a state of the art multi-scale model that can reproduce growth trends as observed in the histology and also to aid our understanding towards the dynamics of this complex process. Real clinical applications of such models are still far from trivial as the current development is relatively at a simple scale. However, the growth trends produced in the presence of a functional endothelium are still sufficiently compelling and demand for further animal studies specifically looking at the functionality of the regenerated endothelium. A three dimensional ISR model, involving re-endothelialization, SMC growth, thrombosis and drug elution from the stent, is also in its final stage of development and that will have the capability to reproduce the growth dynamics of ISR in a more realistic three dimensional space. Once these models are qualitatively and quantitatively validated against porcine in-vivo data, then they can also be applied to patient-specific geometries where they could predict the growth response based on different stent designs and may contribute as a predictive tool in the clinical settings and help in designing better endovascular stents.

### Conclusions

A hypothesis based on our results dictates that a presence of a functional endothelium may be the only regulator that controls the growth of the underlying tissue. But the existence of this functional layer seems to be completely patient or animal specific where blood borne agonists, genetical properties of individuals, ageing and many more so far unknown factors may play an important role [2], [25], [61]. More clinical or experimental investigations are required to obtain animal or patient specific time frames, showing the presence of a healthy endothelium. This will allow us to further validate our current findings. Acquiring such information, however, is far from trivial based on the available knowledge or techniques. To conclude, an ISR response, showing a qualitative agreement with the porcine data (figure 6) [32], has been observed assuming the presence of a functional endothelium. According to our model results, a late endothelial recovery seems to lead towards a restenosis, specially if there is not a fast partial recovery early after stenting. Moreover, if a functional endothelium appears early enough, no ISR is observed, but just some neointima covering the struts as observed in the histological data (unpublished data).
